# Effects of metaphyseal bone tumor removal with preservation of the epiphysis and knee arthroplasty

**DOI:** 10.3892/etm.2014.1744

**Published:** 2014-05-28

**Authors:** PENG ZHANG, FEIFEI FENG, QIQING CAI, WEITAO YAO, SONGTAO GAO, JIAQIANG WANG, XIN WANG

**Affiliations:** 1 Department of Bone and Soft Tissue Cancer, The Affiliated Cancer Hospital of Zhengzhou University, Henan Cancer Hospital, Zhengzhou, Henan 450008, P.R. China; 2 Department of Toxicology, College of Public Health, Zhengzhou University, Zhengzhou, Henan 450001, P.R. China

**Keywords:** metaphyseal bone tumors, epiphysis preservation, physeal distraction, knee arthroplasty, limb length discrepancy

## Abstract

In this study, the effects of surgical removal of malignant metaphyseal bone tumors with epiphysis preservation and knee arthroplasty were analyzed. A total of 15 patients with malignant metaphyseal bone tumors were investigated. Six of these patients underwent epiphyseal preservation surgery with preoperative physeal distraction, termed the physeal distraction (PD) group. Nine patients underwent resection of the knee joint, combined with metal prosthesis transfer, termed the knee arthroplasty (KA) group. Tumor control, limb length discrepancy, range of movement (ROM) of the knee and functional outcome of lower limb [Musculoskeletal Tumor Society (MSTS) score and the Toronto extremity salvage score (TESS)] were assessed for these two groups. All 15 patients were followed-up after the surgery. One patient in the PD group was found to have lung tumor metastasis; however, no local tumor recurrence was found. In the KA group, local tumor recurrence was found in one patient, and lung metastases were observed in two cases postoperatively. The limb length discrepancy in patients of the PD group was 2.58±0.27 cm, which was significantly less compared with that in patients in the KA group (4.01±0.13 cm; P<0.05). In addition, the lower limb knee ROM in patients in the PD group was 127.70±14.63°, which was increased compared to that in patients in the KA group (105.70±15.48°; P<0.05). The mean MSTS score was 86.67% with a mean TESS of 82.33% in patients from the PD group, which showed no significant difference compared with the respective scores for patients in the KA group (P>0.05). Therefore, epiphyseal sparing limb-saving surgeries should be considered for the treatment of malignant metaphyseal bone tumors in children, when certain indications are satisfied.

## Introduction

Malignant bone tumors are the eighth most common type of tumor in children, accounting for 2.4% (1) of all childhood cancers. According to a review conducted by the National Cancer Institute of America, the age-adjusted incidence rate for all bone and joint cancers for all ages and races is 0.9/100,000 individuals per year, and the mortality rate is 0.4/100,000 individuals (2). Osteosarcoma and Ewing’s sarcoma are the two most predominant malignant bone tumors in children and adolescents (3).

Osteosarcoma is derived from primitive bone-forming mesenchymal cells and is the most common primary bone malignancy, accounting for 56% of bone tumors (3). The incidence rate of osteosarcoma for all races and both genders is 4.0 (3.5–4.6 95% confidence interval) for the age range of 0–14 years (per year, per million individuals), and the 10–14-year-old age group has the highest incidence of osteosarcoma, coinciding with the pubertal growth spurt (1).

A total of 75% of malignant bone tumors in children and adolescents occur in the distal femur, near the metaphyseal growth plates (1). The most common strategy for the removal of malignant metaphyseal bone tumors is tumor resection with epiphysis preservation by preoperative physeal distraction, transepiphyseal resection, knee arthroplasty (knee joint resection) and amputation (4–8). Epiphysis preservation with preoperative physeal distraction was first described by Cañadell *et al* (9) in 1994, and may be performed when the tumor has not transgressed the physis and ≥5 mm of normal bone is preserved above the physis on the sagittal section, as determined using magnetic resonance imaging (MRI) (10). Under these conditions, physeal distraction allows separation of the epiphysis from the tumor-bearing metaphysis. However, when the tumor has crossed the physis, it is necessary to perform knee joint resection combined with metal prosthesis transfer, known as knee arthroplasty (11).

In the present study, the effects of metaphyseal bone tumor removal with epiphysis preservation and knee arthroplasty were analyzed by assessing tumor control, limb growth capacity, range of movement of the knee and functional outcomes of the lower limb.

## Patients and methods

### Patients

Between 2007 and 2012, 15 patients with malignant metaphyseal bone tumors underwent tumor resection. Six of these procedures involved physeal distraction and subsequent joint-preserving tumor excision and tumor prosthesis for all patients with knee joint reconstruction, which were transplanted with an allograft or autograft bone. This group was termed the physeal distraction (PD) group; patient information for the PD group is listed in Table I. Nine patients underwent resection of the knee joint combined with metal prosthesis transfer and were labeled the knee arthroplasty (KA) group (Table II). The tumor was located in the distal femur in all 15 patients in the PD and KA groups. The histological diagnosis was osteosarcoma in all patients in the PD group and osteosarcoma in seven and Ewing’s sarcoma in two patients in the KA group.

All patients were administered two cycles of neoadjuvant chemotherapy prior to tumor resection, as per National Comprehensive Cancer Center Network guidelines (12). The protocol included a high dose of methotrexate (8–12 g/m^2^), Adriamycin (60–90 g/m^2^), ifosfamide (2 g/m^2^) and cisplatin (120 g/m^2^) for osteosarcomas.

Written informed consent was obtained from the guardians on the behalf of the participants involved in this study. The Life Sciences Institutional Review Board of Zhengzhou University and The Ethics Committee of Henan Cancer Hospital approved the consent procedures and this study.

### Surgery

#### PD group

**Figure 1:**
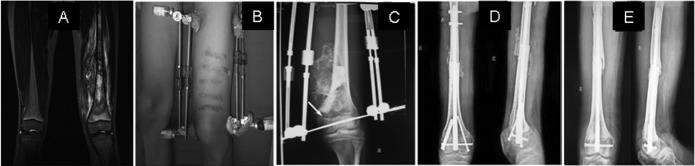
A patient who underwent epiphyseal preservation surgery. (A) A preoperative MRI image shows the tumor not reaching the physis. (B) An external fixator with a T-shaped piece for the epiphysis pins is attached for physeal distraction in the leg of a patient with malignant bone tumor. (C) An X-ray image shows the separation of the epiphysis from the metaphysis (white arrow). (D) An anteroposterior and lateral X-ray image shows the situation of a patient 6 months following tumor resection and reconstruction of the defect with allograft bone graft. (E) An anteroposterior and lateral X-ray image shows the situation of the same patient 2 years following tumor resection and reconstruction of the defect with allograft bone graft. MRI, magnetic resonance imaging.

The indications for physeal distraction were as follows: i) histological examination was used to confirm the presence of a primary bone sarcoma; ii) the tumor was situated in the metaphyseal region and had not metastasized to other organs; iii) the physeal cartilage was intact; iv) the tumor had not transgressed the physis and ≥5 mm of normal bone above the physis was preserved on the sagittal section, as determined using MRI, undertaken prior to treatment (Fig. 1A).

The procedure consisted of three phases. i) Physeal distraction: Two pins were inserted into the epiphysis and another two into the diaphysis, 8–10 cm beyond the tumor. An external monolateral fixator with a T-shaped piece for the epiphysis pins was attached (Fig. 1B). Distraction was performed at a rate of 1–2 mm/day until the physis was disconnected from the epiphysis, as determined by X-ray examination (Fig. 1C). The mean time over which distraction was applied was 12 days. It was possible to carry out this phase while the patient was finishing the course of neoadjuvant chemotherapy. ii) Epiphysis preservation surgery: En-bloc resection was performed, leaving a wide margin, without exposing the metaphyseal surface of the physis. The resected tumor was immediately sent for histological examination. iii) Prosthetic reconstruction with allograft or autograft bone graft: Reconstruction of the bone defect was undertaken as soon as the pathologist reported the absence of tumor at the edges of the resected segment. A bone allograft or autograft was then inserted (Fig. 1D and E).

If tumor cells were found at the physeal edge of the resection, the epiphysis was excised, and the limb was reconstructed using other means (prosthesis or knee arthroplasty).

#### KA group

**Figure 2:**
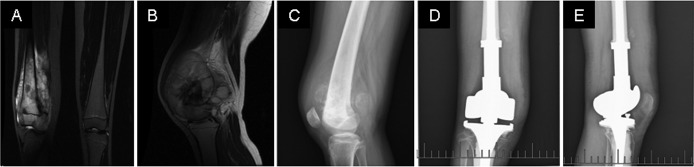
A patient who underwent knee arthroplasty (A and B) Preoperative MRI images show the tumor having crossed the physis. (C) A lateral X-ray image shows the tumor having crossed the physis. (D) An anteroposterior X-ray image shows the situation of the patient 6 months following knee joint and tumor resection, and reconstruction of the defect with metal prosthesis. (E) A lateral X-ray image shows the situation of the same patient 6 months following knee arthroplasty. MRI, magnetic resonance imaging.

A total of 9 patients, in which the metaphyseal bone tumor crossed the physis, as seen on the MRI (Fig. 2A and B) and X-ray (Fig. 2C) images, underwent resection of the knee joint and were outfitted with a metal prosthesis (Fig. 2D and E), a procedure known as knee arthroplasty.

### Postoperative treatment

All patients were intravenously administrated neoadjuvant chemotherapy for four to six cycles following the surgery, depending on the patient response. Patients were allowed to do rehabilitation exercises of active extension and flexion following wound healing.

### Follow-up

Postoperative results of all patients in the two groups were evaluated at a follow-up appointment using knee range of movement (ROM), the Musculoskeletal Tumor Society (MSTS) (13) score and the Toronto extremity salvage score (TESS) (14). In addition, tumor prognosis, length of lower limb and complications, including delayed wound healing, delayed bone union (>12 months with little new bone formation, post-operatively) or non-union (>1 year without new bone formation, post-operatively) were recorded.

### Statistical analysis

Data are expressed as the mean ± the standard error of the mean. GraphPad Software (San Diego, CA, USA) was used for statistical analysis. Significant difference between the two groups with one variant was determined using a Student’s t-test. P<0.05 was considered to indicate a statistically significant difference.

## Results

### Duration of the follow-up time

The follow-up in the PD group was 1–5 years, with a mean duration of 2.5 years, whilst the follow-up time in the KA group was 1–6 years, with a mean duration time of 2.6 years. There was no significant difference in follow-up time between the PD group and the KA group (P>0.05).

### Postoperative results

Five patients in the PD group were alive and disease-free at the last follow-up and one patient succumbed two years following the surgery from lung tumor metastasis. No local tumor recurrence was found, although delayed union occurred in one patient following surgery.

In the KA group, there was local tumor recurrence in one patient, five months following the operation, whereupon the lower limb with the tumor was amputated. Two patients exhibited lung tumor metastasis six months following the surgery. One of the two patients succumbed 27 months following the surgery; however, the other patient was still alive with the tumor at the final follow-up 36 months following the surgery. The metal prosthesis became exposed outside the leg in one patient four months following the procedure, whereupon the lower limb was amputated.

### Growth capacity of the lower limb

**Figure 3:**
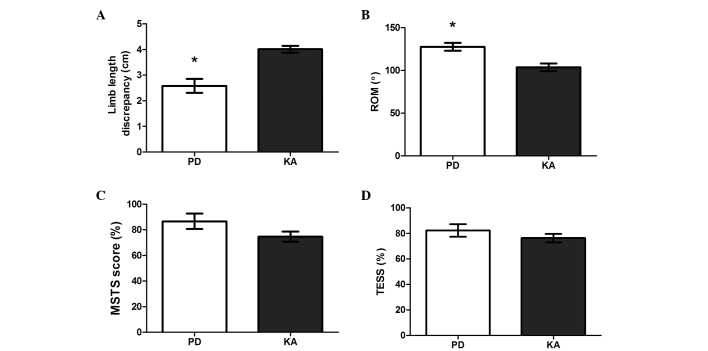
Functional outcome of the lower limb in the two groups. (A) The length discrepancy of the lower limb with the tumor, which was resected, and the other healthy lower limb in patients of the PD group was significantly smaller compared with that in patients in the KA group (^*^P<0.05 versus the KA group). (B) The knee ROM of lower limbs with tumor resection in patients from the PD group was significantly increased, compared with that in patients in the KA group (^*^P<0.05 versus KA group). (C and D) There was no significant difference between the PD and KA groups in MSTS score or TESS results. PD physeal distraction group; KA, knee arthroplasty group; ROM, range of motion; MSTS, Musculoskeletal Tumor Society; TESS, Toronto extremity salvage score.

The length of the lower limb is negatively influenced by epiphysis when children reach adulthood. The length discrepancy between the lower limb that received the surgery and the other healthy lower limb in patients in the PD group was 2.58±0.27 cm, which was significantly smaller compared with the length discrepancy of two lower limbs in patients in the KA group (4.01±0.13 cm) (t=4.691; P=0.009; Fig. 3A).

### Functional outcome of the knee

The knee ROM of the lower limb with tumor resection in patients in the PD group was 127.70±14.63°, which showed an increase compared with that in patients in the KA group (105.70±15.48°) (t=3.723; P=0.020; Fig. 3B). The MSTS score and TESS results of lower limb with tumor resection in patients in the PD group were 86.67±6.06% and 82.33±4.98%, respectively, and 74.67±4.84% and 76.33±3.82% in patients in the KA group, respectively. There was no significant difference between the PD and KA groups with regard to MSTS scores (t=1.671; P=0.170) or TESS (t=1.006; P=0.371) (Fig. 3C and D).

## Discussion

Conservation surgery for malignant bone tumors of the limb is becoming increasingly common due to improvements in diagnostic imaging, the efficacy of chemotherapy and radiotherapy and advances in the reconstruction of bone defects (15). Epiphyseal preservation surgery, knee arthroplasty (knee joint resection) and transepiphyseal resection are common techniques used in malignant bone tumor conservation.

There are a number of advantages of epiphyseal preservation in the removal of malignant bone tumors. Firstly, epiphysis preservation with preoperative physeal distraction may provide a safe margin of resection to prevent tumor reoccurrence. When resecting a tumor, all the malignant tissue must be removed; therefore, in this study, the presence of ≥5 mm of normal bone above the physis was one of the most important indications in determining whether or not to perform physeal distraction. If the tumor is in contact with part of the physis, physeal distraction may only be attempted, and intraoperative histology is recommended (9). If tumor cells are found in the physeal margin of the resection, transepiphyseal resection or knee arthroplasty is the best method of surgical treatment, instead of physeal distraction. When the tumor has crossed the physis, resection requires the loss of the adjacent joint, and knee arthroplasty must be performed as a conservative surgery. In this way, safety is ensured, since all malignant tissue is excised. However, San-Julian *et al* (16) reported promising results of physeal distraction, even if the tumor was in close contact with the physis.

In the present study, five out of six patients in the PD group were alive and disease-free, and there was no local tumor recurrence at last follow-up. The postoperative results demonstrated that the safety margin produced by physeal distraction may ensure complete resection of tumor tissue. In the KA group, the local tumor recurred in one patient, five months following the surgery. This may be as a result of malignant cells in the muscle tissue or fascia around the tumor prior to the knee arthroplasty, which is why it is necessary to make MRI images of tumors and the adjacent tissue and carefully observe them to exclude nearby tumor metastasis. One patient in the PD group and two patients in the KA group were found to have lung metastases, which suggests that these two conservation surgery techniques do not completely prevent metastasis. It is possible that a number of malignant cells had already migrated into the blood or lymph, which were not detected by diagnostic methods prior to tumor resection.

Physeal distraction is safer than transepiphyseal resection since transepiphyseal resection is more difficult to perform on a super complex growth plate with irregular surfaces, and may result in incomplete tumor excision (17). Physeal distraction is performed preoperatively as the first stage of the surgery, with the separation of the growth plate and tumor. Therefore, the tumor may be resected completely by a diaphyseal osteotomy.

In addition, physeal distraction allows for preservation of the epiphysis for limb lengthening in the growing bone of children and adolescents. The epiphysis, the rounded end of a long bone where it joins with the adjacent bone, is responsible for bone lengthening, which is indispensable in growing children. Progressive limb length discrepancies are likely to occur following removal of the growth cartilage. In the present study, the leg length discrepancy in the PD group was significantly decreased compared with that in the KA group (Fig. 3A). This is due to the fact that Cañadell’s technique of preoperative physeal distraction leaves behind a widened boundary of newly formed bone, and the epiphysis is preserved with its regenerative ability, to allow the cells of the germinal layer to grow. Thus, this novel technique of ‘organic’ reconstruction may decrease limb length discrepancies compared with biological reconstruction or reconstruction with metal prosthesis. Langlois and Laville (18) investigated limb lengthening and angular deformations in 15 patients who had undergone physeal distraction surgery, and found that limb length discrepancy and angular deformation may be simultaneously corrected with this novel technique. The authors concluded that physeal distraction does not require osteotomy and respects the vascular supply to the regenerative tissue. In the present study, however, there was still a limb length discrepancy of 2–3 cm in patients in the PD group, even though the discrepancy was smaller compared with that observed with other techniques. Previous studies have demonstrated that there is a close association between the rate of physeal distraction and limb lengthening. De Bastiani *et al* (19) compared the effects of two rates of distraction of the epiphyseal plate and noted that, with rapid distraction at a rate of 1 mm/day for seven days, almost complete ossification of the cartilage was observed after 70 days. By contrast, slow distraction was performed at 0.25 mm every 12 h (0.5 mm/day) for 28 days, whereupon the epiphyseal plate returned to a normal thickness with normal cellular morphology after 70 days, which meant that the epiphyseal plate was able to maintain a normal growth rate. In addition, Pereira *et al* (20) distracted the proximal tibial physis of a rabbit with a rate of distraction of 0.5 mm/day for four weeks, and demonstrated that the proximal tibial growth plate maintained a normal growth rate following slow physeal distraction. In the present study, the physis was distracted at the rate of 1–2 mm/day for seven days. This suggests that rapid distraction may damage the integrity of the growth plate or damage some cells in the germinal layer. In future studies, the rate of distraction should be reduced, in order to explore the implicated mechanism and possibly prevent limb length discrepancies.

Epiphyseal preservation, as a type of limb-saving surgery, is advantageous in terms of preserving knee function. In the present study, the ROM of the knees of patients in the PD group increased, compared with that in patients of the KA group (Fig. 3B), the knee joint in the epiphysis preservation surgery was preserved, allowing the knee to flex at a larger angle. This result is consistent with that of Fang *et al* (21). Other functional results from the present study included a mean MSTS score of 86.67% and a mean TESS of 82.33% in patients in the PD group, which failed to show a significant difference when compared with the scores of patients in the KA group (Fig. 3C and D). In accordance with the results from the present study, previous studies have demonstrated that the functional outcomes of epiphysis preservation surgery are similar to those of other limb-saving techniques (22,23).

Despite these promising results, postoperative complications remain a significant problem with this novel technique. These complications may include delayed union or non-union at the allograft-host junction. To solve this problem, locked plating systems or stronger interlocking intramedullary nails were used in the present study to improve fixation of the allograft to the host bone. Additionally, joint contractures and prosthetic loosening are the main complications of knee arthroplasty; therefore, continued improvements of prostheses are very important.

In conclusion, epiphyseal preservation surgery is an effective limb-saving technique to treat malignant metaphyseal bone tumors in children and adolescents when strict indications are satisfied. The epiphyseal preservation surgery should be considered firstly in this situation, since it results in a smaller limb length discrepancy, larger range of movement of the knee and good functional outcomes of lower limbs. However, when the indications are not satisfied, knee arthroplasty should be performed as a limb-saving surgery in order to completely remove the tumor.

## Figures and Tables

**Table I:** Clinical data of patients in the physeal distraction group.

Patient	Age (years)	Gender	Limb with tumor	Tumor location	Distance of tumor physeal line (mm)	Histological diagnosis	Clinical stage	Duration of distraction (days)
1	12	Male	Left	Distal femur	10	Osteosarcoma	IIB	7
2	14	Male	Left	Distal femur	7	Osteosarcoma	IIB	4
3	13	Female	Right	Distal femur	12	Osteosarcoma	IIB	5
4	9	Male	Left	Distal femur	5	Osteosarcoma	IIA	5
5	11	Male	Right	Distal femur	10	Osteosarcoma	IIB	7
6	13	Female	left	Distal femur	15	Osteosarcoma	IIB	6

**Table II:** Clinical data of patients in the knee arthroplasty group.

Patient	Age (years)	Gender	Limb with tumor	Tumor location	Distance of tumor physeal line (mm)	Histological diagnosis	Clinical stage
1	10	Male	Right	Distal femur	2	Osteosarcoma	IIB
2	13	Female	Left	Distal femur	3	Osteosarcoma	IIA
3	12	Male	Left	Distal femur	3	Ewing’s sarcoma	IIB
4	12	Male	Right	Distal femur	0	Osteosarcoma	IIB
5	14	Male	Right	Distal femur	1	Osteosarcoma	IIB
6	11	Female	Left	Distal femur	3	Ewing’s sarcoma	IIA
7	9	Male	Right	Distal femur	1	Osteosarcoma	IIB
8	16	Female	Left	Distal femur	2	Osteosarcoma	IIB
9	12	Male	Left	Distal femur	3	Osteosarcoma	IIA
